# Building laboratory capacity to combat disease outbreaks in Africa

**DOI:** 10.4102/ajlm.v5i3.579

**Published:** 2016-10-27

**Authors:** Trevor Peter, Mah-Sere Keita, John Nkengasong

**Affiliations:** 1 Clinton Health Access Initiative, Gaborone, Botswana; 2 African Society for Laboratory Medicine, Addis Ababa, Ethiopia; 3 Centers for Disease Control and Prevention, Atlanta, Georgia, United States

The major Ebola outbreak in West Africa in 2014 has brought the world’s attention to the critical demands of health systems strengthening in Africa. Global health responses to outbreaks have never before been tested to the extent driven by Ebola; however, many lessons were learned and countries are actively working on bolstering capacity to detect and respond effectively to Ebola and other outbreaks of global health security importance. Laboratory testing played a key role in the response, and a number of key improvements in laboratory capacity have been made since 2014, with support from African and international partners, including the African Society for Laboratory Medicine, the World Health Organization’s Regional Office for Africa (WHO AFRO) and the United States Centers for Disease Control and Prevention, among others.

Moving forward, the continent needs to set a new trajectory in healthcare development that will prevent future Ebola outbreaks from reaching the 2014 scale, as well as tackle the other disease challenges that Africa disproportionately carries. Cognisant of this and the critical role of laboratory testing in multiple aspects of disease control – surveillance and rapid detection, clinical management and patient care, and the programme management of outbreak responses – this issue of the African Journal for Laboratory Medicine publishes here a set of seminal articles that provide a blueprint for countries and partners on strengthening laboratories in Africa to address the urgent needs of the Global Health Security Agenda and the International Health Regulations.

Kennedy, Wurie and colleagues provide excellent insights into the prior condition of laboratory services in two countries at the focus of the Ebola epidemic, Liberia and Sierra Leone. They highlight conditions that hampered more effective responses, including gaps in human resource capacity, laboratory systems and infrastructure, and argue for national policies, planning, operations and investment that will establish appropriate laboratory institutions and networks supportive of the health systems necessary for laboratory testing in outbreak situations to be impactful. Their observations remind us of familiar challenges; however, in the post-Ebola context their recommendations highlight how common laboratory capacity problems now have new urgency and require innovative solutions.

Philip Onyebujoh from the World Health Organization’s Regional Office for Africa presents a vision for integrating national laboratory networks, surveillance systems and health research institutions to strengthen public health systems. While these are important components of the armoury of national disease programmes, in many African countries these areas operate independently and fail to achieve much-needed synergy. This is a major limitation to countries achieving the Global Health Security Agenda and International Health Regulations requirements and steps need to be taken to address them. Strong leadership, as well as political and financial support, will be required to move swiftly. Close coordination among the key stakeholders will be essential.

The articles by Best, Ondoa and Cowan provide critical new frameworks to address many of the above needs and the tools needed to guide the laboratory strengthening process. Best provides a framework for developing and strengthening functional, tiered laboratory networks, covering each of the systems necessary for optimal network operation and management. This paper also provides a much-needed update on the 2008 Maputo Declaration on Strengthening Laboratory Services by defining a new list of essential laboratory tests required at different levels of the health system to address Global Health Security Agenda priorities. The Maputo Declaration originally established standards for test service delivery that have been adopted across many countries. The update presented here is timely and should become the new standard.

Ondoa presents a quantitative scorecard tool to assess this capacity and, importantly, assess the functionality and readiness of laboratory networks for outbreak situations. This tool is based on the functional, tiered laboratory framework presented by Best and assesses core functions of laboratory networks essential for rapid and effective outbreak responses. The scorecard also provides an innovative outbreak laboratory-readiness scoring system that will be useful for governments, implementing partners, policy makers and donors.

Similarly, Cowan provides a practical framework for regulating the use of diagnostics in outbreak situations. New tests for diseases like Ebola and Zika are often rapidly developed in times of outbreak. Given the urgency of outbreak situations and the need for governments to make rapid decisions on the use of such diagnostic technologies, this guidance is invaluable.

The articles by Perovic, Schultsz and Okeke tackle the looming crisis of antimicrobial resistance. They highlight the critical role of laboratories in antimicrobial resistance detection and containment and note serious deficiencies in current capacity in Africa. In light of this, they provide a practical stepwise approach for implementation of laboratory-based antimicrobial resistance surveillance in African countries. Antimicrobial resistance in Africa is a major but neglected public health priority; the guidance provided in these unique articles is a call to action and provides a foundation for building an effective response.

The articles published in this issue of AJLM are timely and highly relevant as Africa takes important steps toward strengthening its defences against the next outbreak. The tools, guidance and vision they provide should become the blueprint for laboratory capacity necessary to combat disease outbreaks in Africa and for strengthening health security programmes internationally.

